# Potential indicator species of climate changes occurring in Québec, Part 1: the small brown lacewing fly *Micromus posticus* (Walker) (Neuroptera: Hemerobiidae)

**DOI:** 10.3897/BDJ.1.e970

**Published:** 2013-09-16

**Authors:** Laurent LeSage, Karine Savard, Jan Klimaszewski

**Affiliations:** † Agriculture and Agri-Food Canada, Ottawa, Canada; ‡ Laurentian Forestry Centre, Québec, Canada

**Keywords:** Eastern Canada, Québec, Neuroptera, Hemerobiidae, *Micromus posticus*, indicator species, climate change

## Abstract

*Micromus posticus* (Walker) is a small brown lacewing fly rarely collected in Canada and represented in collections by only a limited number of specimens. Indeed, fewer than 50 specimens were captured in Québec and Ontario over the last century, all within a small area delimited by the northern shore of Lake Erie, Ottawa and Montréal. Aylmer, located on the north shore of the Ottawa River, northwest of Ottawa, is a new, most southwestern locality record of this species for Québec. The Aylmer specimens were collected 1-7 days later than any of the known specimens collected elsewhere in Québec or in Ontario, and 16-22 days later than in the neighbouring localities, indicating an apparent phenological shift.

## Introduction

According to the Institut national de santé publique du Québec (INSPQ 2012 - http://www.monclimatmasante.qc.ca/situation-au-québec.aspx), several climate changes are expected in the 21^th^ century in Québec province and adjacent areas: a) winters beginning later and ending earlier, b) winters generally getting milder, c) summers getting longer and hotter, d) thunderstorms becoming more numerous, often with heavier rain, and e) more snow on the ground in the north, and less in the south. Late winters with milder conditions are beneficial for insect species and allow them to be on the wing later than usual during a season, especially during fall. Such late-flying species exist in the family Hemerobiidae (Neuroptera), a family of small soft-bodied insects, often referred to as the brown lacewings. The native *Micromus posticus* (Walker, 1853) belongs to this family. The purpose of this paper is to provide new information on its distribution and new phenology in Québec. This paper is the first in a series in which the senior author is planning to review species that are changing their northern distribution range due to climate warming.

## Materials and methods

*Micromus posticus* was rare in the surveyed area. No specimens were seen or collected from 2007 to 2010. Only three specimens were collected in 2011. Their collection data are given below and in order to compare these captures with specimens collected elsewhere, the data of the latest specimens preserved in public collections in Québec, and in the Canadian National Collection, in Ottawa, are compared in Table 1.

### Habitat

An acre of original mixed forest was continuously monitored from 2007 to 2011 using a black light trap permanently operating from March to December, 10 funnel Lindgren traps installed year round throughout the lot, and 10 yellow pan traps set on the ground since 2007 and maintained year round exactly in the same location during this study. The experimental lot is located on private land in Aylmer, Québec (45° 24' N; 75° 50' W), on the north shore of the Ottawa River, 10.5 km northwest of the national capital Ottawa (Ontario).

The studied area consists of a mixed forest on a limestone outcrop. The red cedar (*Thuya occidentalis*) is the dominant tree but white spruce (*Picea glauca*) and hemlock (*Tsuga canadensis*) being the largest and the oldest on the lot, and approximately 140 years old. The white spruce are at the end of their life span and are often knocked down by the ice storms, heavy snow and strong winds. The hemlock is much more resistant to environmental conditions, are still standing healthy and are less prone to climatic damage. Balsam firs (*Abies balsamea*) are common too but are small, and often rotting inside for an unknown reason. Scattered hardwoods include sugar maple (*Acer saccharum*), bitternut (*Carya cordiformis*), red oak (*Quercus rubra*), aspen (*Populus tremuloides*), American linden (*Tilia americana*) and several small ironwood trees (*Ostrya virginiana*). In the northern portion of the lot, the canopy is more open and the undergrowth of a small area is infested with numerous black buckthorns (*Rhamnus frangula*). Vinegar-tree sumacs (*Rhus typhina*), are confined to the sides of the main entrance and the northern side, established after the entirely forested lot was partially opened for the construction of the house. Tatarian honeysuckle bush (*Lonicera tatarica*) are scattered in an area whereas there are only two individuals of the red-berried elder (*Sambucus pubens*) which badly suffered and almost died during the 2012 long summer drought. The undergrowth is not very rich because the litter is rapidly processed and considerably reduced by earthworms and also due to the closed canopy which does not allow much light to reach the ground. In addition, the soil dries out almost every summer, sometimes deep, supporting only a few herbaceous plants which can resist such adverse ecological conditions. Nevertheless, the large-flowered trillium (*Trillium grandiflorum*) is the most noticeable herbaceous plant among the spring flora. The poison ivy (*Toxicodendron radicans*) resists summer droughts and forms a thick mat behind the house, and the large-leaved aster (*Aster macrophyllus*) is the most common flowering plant among the fall undergrowth.

### Insect collection codens

CEUM – Collection entomologique de l'Université de Montréal. Louise Cloutier

CNC – Canadian National Collection. Laurent LeSage

IMQC – Insectarium de Montréal, Québec. Stéphane LeTirant

LEM – Lyman Entomological Museum. Stéphanie Boucher

LFC – Laurentian Forestry Center, Québec. Jan Klimaszewski

MNC – Museum of Nature of Canada. François Génier

## Taxon treatments

### Micromus posticus

(Walker)

#### Materials

- **Type status:** Other material. **Occurrence:** recordedBy: Reid; individualCount: 1; sex: 1 female; lifeStage: Adult; **Taxon:** scientificName: Micromus posticus (Walker); kingdom: Animalia; phylum: Arthropoda; class: Insecta; order: Neuroptera; family: Hemerobiidae; genus: Micromus; specificEpithet: posticus; scientificNameAuthorship: (Walker); **Location:** continent: North America; country: Canada; countryCode: CA; stateProvince: Ontario; county: Frontenac; municipality: St. Lawrence Islands National Park; locality: Cedar Island; **Identification:** identifiedBy: Jan Klimaszewski; **Event:** eventDate: 31.VIII.1976; year: 1976; month: 8; day: 31; **Record Level:** institutionCode: CNC
- **Type status:** Other material. **Occurrence:** recordedBy: J. Denis; individualCount: 2; sex: 1 male, 1 female; lifeStage: Adult; **Taxon:** scientificName: Micromus posticus (Walker); kingdom: Animalia; phylum: Arthropoda; class: Insecta; order: Neuroptera; family: Hemerobiidae; genus: Micromus; specificEpithet: posticus; scientificNameAuthorship: (Walker); **Location:** continent: North America; country: Canada; countryCode: CA; stateProvince: Ontario; county: Grenville; municipality: near Kemptville; locality: Flint Hill Forest; **Identification:** identifiedBy: Jan Klimaszewski; **Record Level:** institutionCode: JKC
- **Type status:** Other material. **Occurrence:** recordedBy: S. & J. Peck; individualCount: 1; sex: 1 male; lifeStage: Adult; **Taxon:** scientificName: Micromus posticus (Walker); kingdom: Animalia; phylum: Arthropoda; class: Insecta; order: Neuroptera; family: Hemerobiidae; genus: Micromus; specificEpithet: posticus; scientificNameAuthorship: (Walker); **Location:** continent: North America; country: Canada; countryCode: CA; stateProvince: Ontario; county: Lanark; municipality: near Carleton Place; **Identification:** identifiedBy: Jan Klimaszewski; **Record Level:** institutionCode: JKC
- **Type status:** Other material. **Occurrence:** recordedBy: S. Peck; individualCount: 1; sex: 1 male; lifeStage: Adult; **Taxon:** scientificName: Micromus posticus (Walker); kingdom: Animalia; phylum: Arthropoda; class: Insecta; order: Neuroptera; family: Hemerobiidae; genus: Micromus; specificEpithet: posticus; scientificNameAuthorship: (Walker); **Location:** continent: North America; country: Canada; countryCode: CA; stateProvince: Ontario; county: Leeds; municipality: Chaffeys Locks; locality: Queen's University Biological Station; **Identification:** identifiedBy: Jan Klimaszewski; **Record Level:** institutionCode: JKC
- **Type status:** Other material. **Occurrence:** recordedBy: J.R. Vockeroth; individualCount: 2; sex: 1 male, 1 female; lifeStage: Adult; **Taxon:** scientificName: Micromus posticus (Walker); kingdom: Animalia; phylum: Arthropoda; class: Insecta; order: Neuroptera; family: Hemerobiidae; genus: Micromus; specificEpithet: posticus; scientificNameAuthorship: (Walker); **Location:** continent: North America; country: Canada; countryCode: CA; stateProvince: Ontario; county: Leeds; municipality: Lyndhurst; **Identification:** identifiedBy: Jan Klimaszewski; **Event:** eventDate: 10.VII.1958; year: 1958; month: 7; day: 10; **Record Level:** institutionCode: CNC
- **Type status:** Other material. **Occurrence:** recordedBy: Reid; individualCount: 1; sex: 1 male; lifeStage: Adult; **Taxon:** scientificName: Micromus posticus (Walker); kingdom: Animalia; phylum: Arthropoda; class: Insecta; order: Neuroptera; family: Hemerobiidae; genus: Micromus; specificEpithet: posticus; scientificNameAuthorship: (Walker); **Location:** continent: North America; country: Canada; countryCode: CA; stateProvince: Ontario; county: Leeds; municipality: St. Lawrence Islands National Park; locality: Aubrey Island; **Identification:** identifiedBy: Jan Klimaszewski; **Event:** eventDate: 15.IX.1976; year: 1976; month: 9; day: 15; **Record Level:** institutionCode: CNC
- **Type status:** Other material. **Occurrence:** recordedBy: Reid; individualCount: 1; sex: 1 female; lifeStage: Adult; **Taxon:** scientificName: Micromus posticus (Walker); kingdom: Animalia; phylum: Arthropoda; class: Insecta; order: Neuroptera; family: Hemerobiidae; genus: Micromus; specificEpithet: posticus; scientificNameAuthorship: (Walker); **Location:** continent: North America; country: Canada; countryCode: CA; stateProvince: Ontario; county: Leeds; municipality: St. Lawrence Islands National Park; locality: McDonald Island; **Identification:** identifiedBy: Jan Klimaszewski; **Event:** eventDate: 10.IX.1976; year: 1976; month: 9; day: 10; **Record Level:** institutionCode: CNC
- **Type status:** Other material. **Occurrence:** recordedBy: Reid; individualCount: 2; sex: 1 male, 1 female; lifeStage: Adult; **Taxon:** scientificName: Micromus posticus (Walker); kingdom: Animalia; phylum: Arthropoda; class: Insecta; order: Neuroptera; family: Hemerobiidae; genus: Micromus; specificEpithet: posticus; scientificNameAuthorship: (Walker); **Location:** continent: North America; country: Canada; countryCode: CA; stateProvince: Ontario; county: Leeds; municipality: St. Lawrence Islands National Park; locality: Thwartway Island; **Identification:** identifiedBy: Jan Klimaszewski; **Event:** eventDate: 19.VIII.1976; year: 1976; month: 8; day: 19; **Record Level:** institutionCode: CNC
- **Type status:** Other material. **Occurrence:** recordedBy: Reid; individualCount: 1; sex: 1 male; lifeStage: Adult; **Taxon:** scientificName: Micromus posticus (Walker); kingdom: Animalia; phylum: Arthropoda; class: Insecta; order: Neuroptera; family: Hemerobiidae; genus: Micromus; specificEpithet: posticus; scientificNameAuthorship: (Walker); **Location:** continent: North America; country: Canada; countryCode: CA; stateProvince: Ontario; county: Leeds; municipality: St. Lawrence Islands National Park; locality: Mulcaster Island; **Identification:** identifiedBy: Jan Klimaszewski; **Record Level:** institutionCode: JKC
- **Type status:** Other material. **Occurrence:** recordedBy: W.L. Putman; individualCount: 1; sex: 1 female; lifeStage: Adult; **Taxon:** scientificName: Micromus posticus (Walker); kingdom: Animalia; phylum: Arthropoda; class: Insecta; order: Neuroptera; family: Hemerobiidae; genus: Micromus; specificEpithet: posticus; scientificNameAuthorship: (Walker); **Location:** continent: North America; country: Canada; countryCode: CA; stateProvince: Ontario; county: Lincoln; municipality: Niagara-on-the-Lake; **Identification:** identifiedBy: Jan Klimaszewski; **Event:** eventDate: 17.X.1932; year: 1932; month: 10; day: 17; **Record Level:** institutionCode: CNC
- **Type status:** Other material. **Occurrence:** recordedBy: W.L. Putman; individualCount: 1; sex: 1 female; lifeStage: Adult; **Taxon:** scientificName: Micromus posticus (Walker); kingdom: Animalia; phylum: Arthropoda; class: Insecta; order: Neuroptera; family: Hemerobiidae; genus: Micromus; specificEpithet: posticus; scientificNameAuthorship: (Walker); **Location:** continent: North America; country: Canada; countryCode: CA; stateProvince: Ontario; county: Lincoln; municipality: Vineland; **Identification:** identifiedBy: Jan Klimaszewski; **Event:** eventDate: 14.IX.1929; year: 1929; month: 9; day: 14; **Record Level:** institutionCode: CNC
- **Type status:** Other material. **Occurrence:** recordedBy: J.A. Hall; individualCount: 1; lifeStage: Adult; **Taxon:** scientificName: Micromus posticus (Walker); kingdom: Animalia; phylum: Arthropoda; class: Insecta; order: Neuroptera; family: Hemerobiidae; genus: Micromus; specificEpithet: posticus; scientificNameAuthorship: (Walker); **Location:** continent: North America; country: Canada; countryCode: CA; stateProvince: Ontario; county: Simcoe; municipality: Norfolk; **Identification:** identifiedBy: Jan Klimaszewski; **Event:** eventDate: 23.VII.1936; year: 1936; month: 7; day: 23; **Record Level:** institutionCode: CNC
- **Type status:** Other material. **Occurrence:** recordedBy: J.R. Vockeroth; individualCount: 1; sex: 1 male; lifeStage: Adult; **Taxon:** scientificName: Micromus posticus (Walker); kingdom: Animalia; phylum: Arthropoda; class: Insecta; order: Neuroptera; family: Hemerobiidae; genus: Micromus; specificEpithet: posticus; scientificNameAuthorship: (Walker); **Location:** continent: North America; country: Canada; countryCode: CA; stateProvince: Ontario; municipality: Ottawa; **Identification:** identifiedBy: Jan Klimaszewski; **Event:** eventDate: 18.X.1961; year: 1961; month: 10; day: 18; **Record Level:** institutionCode: CNC
- **Type status:** Other material. **Occurrence:** recordedBy: J.R. Vockeroth; individualCount: 1; lifeStage: Adult; **Taxon:** scientificName: Micromus posticus (Walker); kingdom: Animalia; phylum: Arthropoda; class: Insecta; order: Neuroptera; family: Hemerobiidae; genus: Micromus; specificEpithet: posticus; scientificNameAuthorship: (Walker); **Location:** continent: North America; country: Canada; countryCode: CA; stateProvince: Ontario; municipality: Ottawa; **Identification:** identifiedBy: Jan Klimaszewski; **Event:** eventDate: 27.VIII.1961; year: 1961; month: 8; day: 27; **Record Level:** institutionCode: CNC
- **Type status:** Other material. **Occurrence:** recordedBy: S. Peck; individualCount: 1; sex: 1 male; lifeStage: Adult; **Taxon:** scientificName: Micromus posticus (Walker); kingdom: Animalia; phylum: Arthropoda; class: Insecta; order: Neuroptera; family: Hemerobiidae; genus: Micromus; specificEpithet: posticus; scientificNameAuthorship: (Walker); **Location:** continent: North America; country: Canada; countryCode: CA; stateProvince: Ontario; municipality: Rideau Ferry; locality: Big Rideau Lake; **Identification:** identifiedBy: Jan Klimaszewski; **Record Level:** institutionCode: JKC
- **Type status:** Other material. **Occurrence:** recordedBy: B. Bendel; individualCount: 1; sex: 1 female; lifeStage: Adult; **Taxon:** scientificName: Micromus posticus (Walker); kingdom: Animalia; phylum: Arthropoda; class: Insecta; order: Neuroptera; family: Hemerobiidae; genus: Micromus; specificEpithet: posticus; scientificNameAuthorship: (Walker); **Location:** continent: North America; country: Canada; countryCode: CA; stateProvince: Québec; county: Beauharnois; municipality: Beauharnois; **Identification:** identifiedBy: Jan Klimaszewski; **Record Level:** institutionCode: LEM
- **Type status:** Other material. **Occurrence:** recordedBy: L. LeSage; individualCount: 1; sex: 1 male; lifeStage: Adult; **Taxon:** scientificName: Micromus posticus (Walker); kingdom: Animalia; phylum: Arthropoda; class: Insecta; order: Neuroptera; family: Hemerobiidae; genus: Micromus; specificEpithet: posticus; scientificNameAuthorship: (Walker); **Location:** continent: North America; country: Canada; countryCode: CA; stateProvince: Québec; county: Gatineau; municipality: Aylmer; verbatimLocality: Klock road; verbatimCoordinates: 45 24 N 75 50 W; verbatimCoordinateSystem: degrees minutes; **Identification:** identifiedBy: Jan Klimaszewski; **Event:** eventDate: 8.XI.2011; year: 2011; month: 11; day: 8; habitat: resting on the wall siding of a house surrounded by mixed forest; **Record Level:** institutionCode: CNC
- **Type status:** Other material. **Occurrence:** recordedBy: L. LeSage; individualCount: 2; sex: 1 male, 1 female; lifeStage: Adult; **Taxon:** scientificName: Micromus posticus (Walker); kingdom: Animalia; phylum: Arthropoda; class: Insecta; order: Neuroptera; family: Hemerobiidae; genus: Micromus; specificEpithet: posticus; scientificNameAuthorship: (Walker); **Location:** continent: North America; country: Canada; countryCode: CA; stateProvince: Québec; county: Gatineau; municipality: Aylmer; verbatimLocality: Klock road; verbatimCoordinates: 45 24 N 75 50 W; verbatimCoordinateSystem: degrees minutes; **Identification:** identifiedBy: Jan Klimaszewski; **Event:** eventDate: 2.XI.2011; year: 2011; month: 11; day: 2; habitat: resting on the wall siding of a house surrounded by mixed forest; **Record Level:** institutionCode: CNC
- **Type status:** Other material. **Occurrence:** recordedBy: J.R. Vockeroth; individualCount: 1; sex: 1 female; lifeStage: Adult; **Taxon:** scientificName: Micromus posticus (Walker); kingdom: Animalia; phylum: Arthropoda; class: Insecta; order: Neuroptera; family: Hemerobiidae; genus: Micromus; specificEpithet: posticus; scientificNameAuthorship: (Walker); **Location:** continent: North America; country: Canada; countryCode: CA; stateProvince: Québec; county: Gatineau; municipality: Old Chelsea; **Identification:** identifiedBy: Jan Klimaszewski; **Event:** eventDate: 17.X.1961; year: 1961; month: 10; day: 17; **Record Level:** institutionCode: CNC
- **Type status:** Other material. **Occurrence:** recordedBy: D.N. Duffy; individualCount: 1; sex: 1 female; lifeStage: Adult; **Taxon:** scientificName: Micromus posticus (Walker); kingdom: Animalia; phylum: Arthropoda; class: Insecta; order: Neuroptera; family: Hemerobiidae; genus: Micromus; specificEpithet: posticus; scientificNameAuthorship: (Walker); **Location:** continent: North America; country: Canada; countryCode: CA; stateProvince: Québec; county: Île-de-Montréal; municipality: Baie-d'Urfé; **Identification:** identifiedBy: Jan Klimaszewski; **Record Level:** institutionCode: LEM
- **Type status:** Other material. **Occurrence:** recordedBy: E.A. Monroe; individualCount: 1; sex: 1 female; lifeStage: Adult; **Taxon:** scientificName: Micromus posticus (Walker); kingdom: Animalia; phylum: Arthropoda; class: Insecta; order: Neuroptera; family: Hemerobiidae; genus: Micromus; specificEpithet: posticus; scientificNameAuthorship: (Walker); **Location:** continent: North America; country: Canada; countryCode: CA; stateProvince: Québec; county: Île-de-Montréal; municipality: Montréal; **Identification:** identifiedBy: Jan Klimaszewski; **Record Level:** institutionCode: LEM
- **Type status:** Other material. **Occurrence:** recordedBy: J.A. Garland; individualCount: 6; sex: 2 males, 4 females; lifeStage: Adult; **Taxon:** scientificName: Micromus posticus (Walker); kingdom: Animalia; phylum: Arthropoda; class: Insecta; order: Neuroptera; family: Hemerobiidae; genus: Micromus; specificEpithet: posticus; scientificNameAuthorship: (Walker); **Location:** continent: North America; country: Canada; countryCode: CA; stateProvince: Québec; county: Île-de-Montréal; municipality: Sainte-Anne-de-Bellevue; **Identification:** identifiedBy: Jan Klimaszewski; **Record Level:** institutionCode: LEM
- **Type status:** Other material. **Occurrence:** recordedBy: M. Javahary; individualCount: 1; sex: 1 female; lifeStage: Adult; **Taxon:** scientificName: Micromus posticus (Walker); kingdom: Animalia; phylum: Arthropoda; class: Insecta; order: Neuroptera; family: Hemerobiidae; genus: Micromus; specificEpithet: posticus; scientificNameAuthorship: (Walker); **Location:** continent: North America; country: Canada; countryCode: CA; stateProvince: Québec; county: Île-de-Montréal; municipality: Sainte-Anne-de-Bellevue; **Identification:** identifiedBy: Jan Klimaszewski; **Event:** eventDate: 1.XI.1983; **Record Level:** institutionCode: LEM
- **Type status:** Other material. **Occurrence:** recordedBy: R.L. Manuel; individualCount: 1; sex: 1 male; lifeStage: Adult; **Taxon:** scientificName: Micromus posticus (Walker); kingdom: Animalia; phylum: Arthropoda; class: Insecta; order: Neuroptera; family: Hemerobiidae; genus: Micromus; specificEpithet: posticus; scientificNameAuthorship: (Walker); **Location:** continent: North America; country: Canada; countryCode: CA; stateProvince: Québec; county: Île-de-Montréal; municipality: Sainte-Anne-de-Bellevue; **Identification:** identifiedBy: Jan Klimaszewski; **Record Level:** institutionCode: LEM
- **Type status:** Other material. **Occurrence:** recordedBy: A.C. Sheppard; individualCount: 5; sex: 2 males, 3 females; lifeStage: Adult; **Taxon:** scientificName: Micromus posticus (Walker); kingdom: Animalia; phylum: Arthropoda; class: Insecta; order: Neuroptera; family: Hemerobiidae; genus: Micromus; specificEpithet: posticus; scientificNameAuthorship: (Walker); **Location:** continent: North America; country: Canada; countryCode: CA; stateProvince: Québec; county: Île-Jésus; municipality: Laval; **Identification:** identifiedBy: Jan Klimaszewski; **Record Level:** institutionCode: LEM
- **Type status:** Other material. **Occurrence:** recordedBy: A.T. Finnamore; individualCount: 4; sex: 1 male, 3 females; lifeStage: Adult; **Taxon:** scientificName: Micromus posticus (Walker); kingdom: Animalia; phylum: Arthropoda; class: Insecta; order: Neuroptera; family: Hemerobiidae; genus: Micromus; specificEpithet: posticus; scientificNameAuthorship: (Walker); **Location:** continent: North America; country: Canada; countryCode: CA; stateProvince: Québec; county: Rouville; municipality: Mont-Saint-Hilaire; **Identification:** identifiedBy: Jan Klimaszewski; **Record Level:** institutionCode: LEM
- **Type status:** Other material. **Occurrence:** recordedBy: B. Landry; individualCount: 1; sex: 1 male; lifeStage: Adult; **Taxon:** scientificName: Micromus posticus (Walker); kingdom: Animalia; phylum: Arthropoda; class: Insecta; order: Neuroptera; family: Hemerobiidae; genus: Micromus; specificEpithet: posticus; scientificNameAuthorship: (Walker); **Location:** continent: North America; country: Canada; countryCode: CA; stateProvince: Québec; county: Terrebonne; municipality: Sainte-Thérèse; **Identification:** identifiedBy: Jan Klimaszewski; **Event:** eventDate: 30.V.1985; year: 1985; month: 5; day: 30; **Record Level:** institutionCode: JKC
- **Type status:** Other material. **Occurrence:** recordedBy: R.L. Manuel; individualCount: 1; sex: 1 male; lifeStage: Adult; **Taxon:** scientificName: Micromus posticus (Walker); kingdom: Animalia; phylum: Arthropoda; class: Insecta; order: Neuroptera; family: Hemerobiidae; genus: Micromus; specificEpithet: posticus; scientificNameAuthorship: (Walker); **Location:** continent: North America; country: Canada; countryCode: CA; stateProvince: Québec; county: Vaudreuil; municipality: Île-Perrot; **Identification:** identifiedBy: Jan Klimaszewski; **Record Level:** institutionCode: LEM
- **Type status:** Other material. **Occurrence:** recordedBy: V.R. Vickery; individualCount: 1; sex: 1 female; lifeStage: Adult; **Taxon:** scientificName: Micromus posticus (Walker); kingdom: Animalia; phylum: Arthropoda; class: Insecta; order: Neuroptera; family: Hemerobiidae; genus: Micromus; specificEpithet: posticus; scientificNameAuthorship: (Walker); **Location:** continent: North America; country: Canada; countryCode: CA; stateProvince: Québec; county: Vaudreuil; municipality: Île-Perrot; locality: Pincourt; **Identification:** identifiedBy: Jan Klimaszewski; **Record Level:** institutionCode: LEM

#### Diagnosis

**Figure 1.**
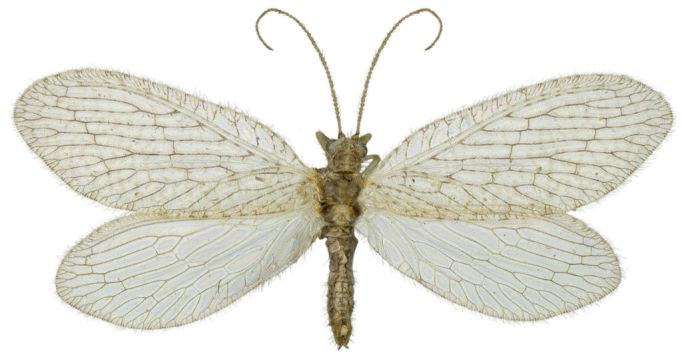
*Micromus posticus* (Walker): habitus dorsal view.

Body length: 6-7.5 mm; wing span: 10-20 mm. Head, body and appendages yellow to brownish, wings brownish (Fig. 1). Fore wing with radial sector (R_s_) bearing 4 main veins; distance between the inner gradate cross-veins not more than their length (Fig. 2a); hind wing hyaline without maculation (Fig. 2b). Male ectoproct with a styliform and slightly dorsally produced ventral lobe (Fig. 2c); gonarcus with two spine-like anterior structures with and lateral triangular indentation and a broadly oval dorsal projection (Fig. 2d). For more details and extensive description see Klimaszewski and Kevan (1988).

#### Discussion

The status of so called "rare" species is often difficult to establish in fall occurring species because very little collecting is made in September, and almost never in November or December. Consequently, a proportion of the apparent rarity of a species may not be real but due to the lack of appropriate collecting events. Nevertheless, *Micromus posticus* seems truly rare since only 49 specimens (Ontario, 23 specimens; Québec, 26 specimens) were collected in these two provinces over the last 100+ years, on the basis of specimens housed in public collections (Table 1).

**Figure 3.**
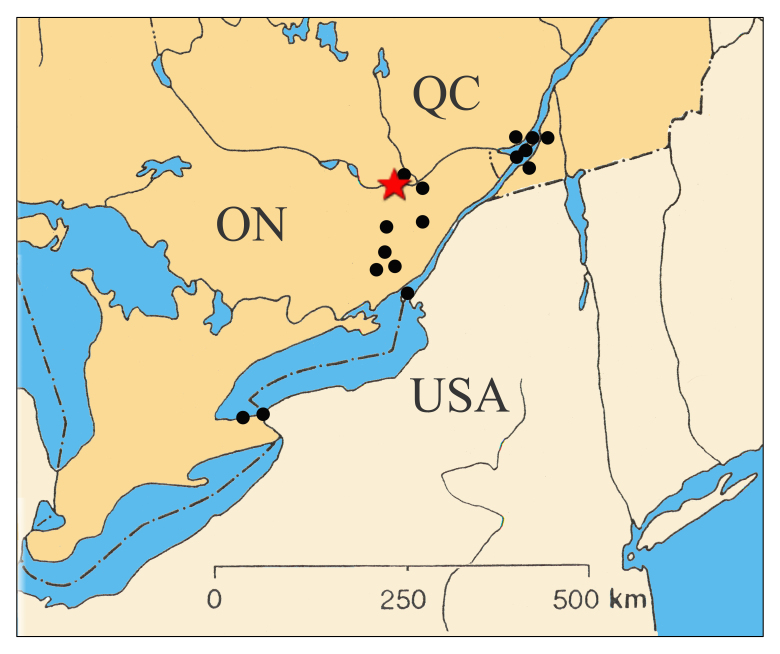
Distribution map of *Micromus posticus* in Canada - black dots; red star indicates the Aylmer record.

Although *Micromus posticus* has been reported from British Columbia and Yukon, the majority of the known collection sites, are located along the Great Lakes - St. Lawrence River system (Klimaszewski and Kevan 1988). In Ontario, all known localities are spread between Lake Erie and Ottawa. In Québec, the known distribution of this species is restricted southwest to the vicinity of Aylmer, and eastwards to around Montréal Island (Fig. 3). Aylmer represents a new locality record for Québec.

The collection dates on the 2^nd^ and 8^th^ November for Aylmer specimens constitute new collecting records for this species. In comparison, the latest previously known specimen was collected on November 1st, 1983 (Table 1). However, it must be pointed out that its collection locality, Sainte-Anne-de-Bellevue, is 148 km east of Aylmer and represents on average warmer area. The latest capture, in Old Chelsea, a village 12 km north of Aylmer, took place on October 17. In Ottawa, 11.5 southeast of Aylmer, the latest known specimens was captured on October 15, 1961. In conclusion, our collecting of *Micromus posticus* in Aylmer, November, was 1-7 days later than any of the known specimens collected from Québec and 16-22 days later than those collected in the neighbouring localities. According to Woodall et al. (2009), the northward migration of trees in the eastern United States is currently underway. In regards to insects, the relaxed cold limitations should favour some pests (Diffenbaugh et al. 2008), but native species should be favoured as well. If this anticipated scenario of species changing their distribution limits northwards continues, *Micromus posticus* will fly over the latitudes of Aylmer or Montréal more often and will get permanently established there in a near future.

## Supplementary Material

XML Treatment for Micromus posticus

## Figures and Tables

**Figure 2a.**
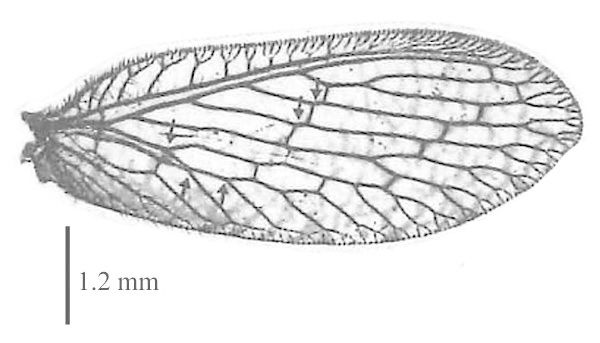
fore wing

**Figure 2b.**
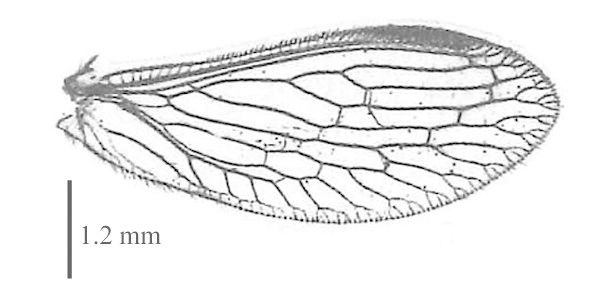
hind wing

**Figure 2c.**
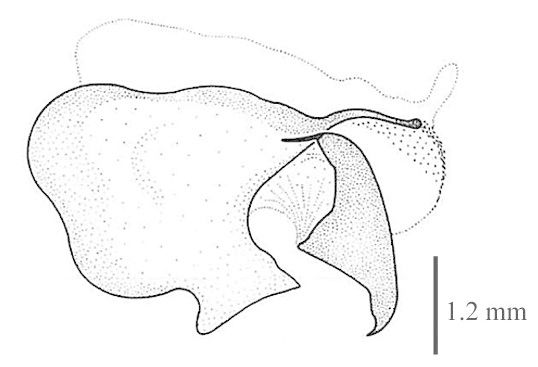
male ectoproct, lateral view

**Figure 2d.**
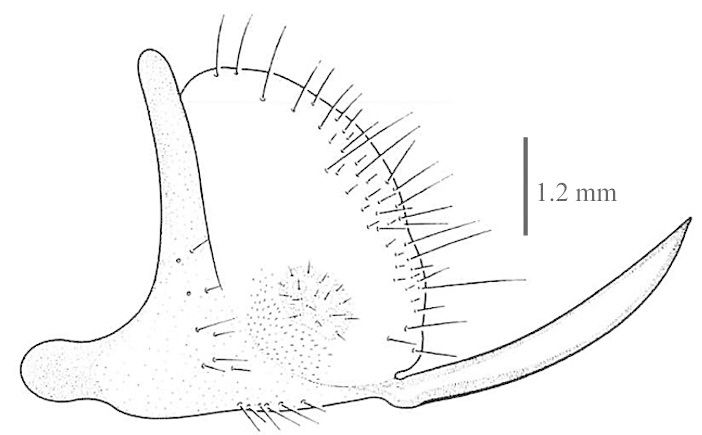
male gonarcus, lateral view

**Table 1.** Summary of collections examined for *Micromus posticus*. Localities, collection dates, and collectors include only the latest specimens found in relevant collections.

Collection	Province	Locality	Date	Collector	Specimens
**CEUM**	**QC**	x	x	x	None
**LFC**	**QC**	x	x	x	None
**CNC**	**QC**	Old Chelsea	17.X.1961	J.R. Vockeroth	1
**CNC**	**ON**	Ottawa	18.X.1961	J.R. Vockeroth	1
**IMQC**	**QC**	x	x	x	None
**LEM**	**QC**	Sainte-Anne-de-Bellevue	1.XI.1983	M. Javahary	1
**MNC**	**QC**	x	x	x	None
